# Circ_0092367 Inhibits EMT and Gemcitabine Resistance in Pancreatic Cancer via Regulating the miR-1206/ESRP1 Axis

**DOI:** 10.3390/genes12111701

**Published:** 2021-10-26

**Authors:** Shuo Yu, Min Wang, Hang Zhang, Xingjun Guo, Renyi Qin

**Affiliations:** Department of Biliary-Pancreatic Surgery, Affiliated Tongji Hospital, Tongji Medical College, Huazhong University of Science and Technology, Wuhan 430030, China; shuoyuys@163.com (S.Y.); minwangmw@126.com (M.W.); hangzhanghz@163.com (H.Z.); xingjunguoxj@163.com (X.G.)

**Keywords:** circular RNA, gemcitabine, EMT, pancreatic cancer

## Abstract

Gemcitabine is the first-line treatment for patients with pancreatic cancer (PC), yet most patients develop resistance to gemcitabine. Recent studies showed that circular RNAs (circRNAs) have important regulatory roles in PC progression and chemoresistance. In this study, the ability of circRNA circ_0092367 to enhance gemcitabine efficacy was tested and the underlying molecular mechanism of circ_0092367 was investigated. The expression levels of circ_0092367, miR-1206, and ESRP1 were measured using qRT-PCR experiments. The effects of circ_0092367, miR-1206, and ESRP1 on PC cell lines exposed to gemcitabine were examined by CCK-8 assays. We performed luciferase assays to determine the relationship between circ_0092367 and miR-1206 and between miR-1206 and ESRP1. We demonstrated that circ_0092367 was significantly downregulated in PC tissues and cell lines, and a high expression of circ_0092367 was associated with improved survival in patients with PC. Gain- and loss-of-function assays revealed that circ_0092367 inhibited epithelial–mesenchymal transition (EMT) phenotypes and sensitized PC cells to gemcitabine treatment in vitro and in vivo. Cytoplasmic circ_0092367 could directly repress the levels of miR-1206 and thus upregulate the expression of ESRP1, thereby inhibiting EMT and enhancing the sensitivity of PC cells to gemcitabine treatment. Our findings show that circ_0092367 plays a crucial role in sensitizing PC cells to gemcitabine by modulating the miR-1206/ESRP1 axis, highlighting its potential as a valuable therapeutic target in PC patients.

## 1. Introduction

Pancreatic cancer (PC) is a highly aggressive human malignancy, and the overall 5-year survival rate of patients with PC is 9% [1]. Chemotherapy is an important part of multimodality PC treatment. Gemcitabine is a first-line drug approved for the treatment of PC [2]. However, chemoresistance seriously limits the effectiveness of chemotherapy. Therefore, the identification of genetic and epigenetic mechanisms involved in chemoresistance is critical for developing effective treatments for PC.

The epithelial to mesenchymal transition (EMT) is considered a contributing factor that activates signaling pathways governing cancer chemoresistance [3]. Indeed, PC chemoresistance has been associated with mesenchymal characteristics [4]. Previous studies have indicated that epithelial splicing regulatory protein 1 (ESRP1) negatively regulates EMT in breast cancer, PC, oral squamous cell carcinoma, and non-small cell lung cancer [5,6,7,8]. However, no study has reported whether ESRP1 can affect PC chemoresistance.

MicroRNAs (miRNAs) are short, non-protein-coding RNAs that bind to the sequence in the 3′-untranslated regions (3′-UTRs) of target mRNAs and regulate gene expression [9]. Some miRNAs can suppress or promote EMT and chemoresistance [10,11]. Circular RNAs (circRNAs) are non-coding RNA molecules that form a closed-loop structure [12]. Importantly, circRNAs function as either tumor suppressors or oncogenes in many cancers and mediate various cellular processes, including EMT and chemoresistance [12]. Growing evidence demonstrates that circRNAs can serve as miRNA sponges, thereby regulating the expression of the target genes of miRNAs [12]. However, those circRNAs involved in PC chemoresistance remain largely unknown. Human circ_0092367 is transcribed from the *SNORD116-14* gene (ENSG00000206621). Although circ_0092367 was downregulated in PC samples compared with normal samples [13], the roles and mechanisms of circ_0092367 in EMT and PC chemoresistance are poorly understood.

In this study, we attempted to discover the contributions and mechanisms of circ_0092367 to the sensitivity of PC to gemcitabine. We demonstrated that circ_0092367 significantly sensitized PC cells to gemcitabine treatment by regulating the miR-1206/ESRP1 axis. Our results revealed a mechanism by which PC evades drug therapy, and thus provided a potential target for PC treatment.

## 2. Materials and Methods

### 2.1. Tumor Specimens

A total of 40 PC tissues and their adjacent normal tissues were collected from patients receiving surgery in Affiliated Tongji Hospital, Tongji Medical College, Huazhong University of Science and Technology, China. All patients had not received preoperative chemotherapy treatment. The clinical and pathological characteristics were obtained from medical records. This study was approved by the Research Ethics Committee of Tongji Medical College, Huazhong University of Science and Technology (TJ-IRB20190418). Each patient gave written informed consent to participate in our study.

### 2.2. Cell Lines and Transfection

We obtained four different PC cell lines (AsPC-1, BxPC-3, SW-1990, and PaCa-2) and a normal pancreatic epithelial cell line (HPDE6-C7) from the ATCC (Manassas, VA, USA). Cells were maintained in RPMI-1640 medium (Invitrogen, Carlsbad, CA, USA) supplemented with 10% fetal bovine serum (Invitrogen). A gemcitabine-resistant cell line, PaCa-2/Gemcitabine, was generated by incubating the parental PaCa-2 cell line with increasing concentrations of gemcitabine, as previously reported [14]. The human embryonic kidney 293 (HEK293) cell line was purchased from ATCC and cultured in DMEM medium (Invitrogen) supplemented with 10% FBS. All cells were incubated in 5% CO_2_ at 37 °C.

Lipofectamine 3000 reagent (Invitrogen) was used to transfect PC cell lines with the miR-1206 mimic and miR-1206 inhibitor (Ribobio, Guangzhou, China). In addition, PC cells were transiently transfected with pCMV6-ESRP1 or with an empty vector (OriGene, Rockville, MD, USA) using the Lipofectamine 3000 reagent (Invitrogen) according to the manufacturer’s instructions.

The circ_0092367-expressing vector was custom constructed by Geneseed Biotech (Guangzhou, China). In brief, the full length of human circ_0092367 was inserted into a pLCDH-ciR lentiviral expression vector (Geneseed Biotech) using the following primers (hsa_circ_0092367-F: 5′-CCGGAATTCTGAAATATGCTATCTTACAGTGGATCGATGATGACTTCCATAT-3′; hsa_circ_0092367-R: 5′-CGCGGATCCTCAAGAAAAAATATATTCACTGGACCTCAGTTCGACGAGAATG-3′).

The shRNA against circ_0092367 (circ_0092367 siRNA: 5′-ATCGATGCATTTGCAGAAACA-3′) and a negative control shRNA-scramble (scramble siRNA: 5′-TTCTCCGAACGTGTCACGT-3′) were synthesized and cloned into pLent-U6-GFP-Puro vectors (Mailgene biosciences, Beijing, China) to generate a shRNA lentivector targeting circ_0092367 and a control shRNA lentivector, named LV-circ_0092367 and LV-scramble, respectively.

We established PaCa-2 cells that stably overexpress circ_0092367 (or circ_0092367-knockdown stable AsPC-1 cell lines) by transfecting the circ_0092367-expressing vector (or LV-circ_0092367) with packaging plasmids (psPAX2 and pMD2.G, Geneseed Biotech) into human embryonic kidney 293 (HEK293) cells using Lipofectamine 3000 (Invitrogen). After cells were cultured for 48 h, the packaged lentiviruses were harvested. Finally, PaCa-2 cells were infected with the virus and selected with 0.5 μg/mL of puromycin (Sigma-Aldrich, St. Louis, MO, USA).

### 2.3. RNA Extraction and qRT-PCR

Using TRIzol reagent (Invitrogen), we isolated total RNA from tissues and cells. Reverse transcription was conducted using the PrimeScript RT Master Mix (Takara, Dalian, China). Total RNA was treated with RNase R (Geneseed, Guangzhou, China) for 30 min at 37 °C. Cytoplasmic or nuclear RNA was isolated using a Nuclear/Cytoplasmic Isolation kit (BioVision, San Francisco, CA, USA). Quantitative PCR was carried out using SYBR Green Real-time PCR Master Mix (Toyobo, Osaka, Japan). The PCR primers were obtained from Ribobio (China). The mirVanaTM qRT-PCR miRNA Detection Kit (Ambion, Austin, TX, USA) was utilized to detect the miR-1206 expression. U6 was used as the reference.

The primer sequences used were as follows: circ_0092367-F: 5′-TCCTCATTTGCAGGGATGGAG-3′; circ_0092367-R: 5′-TCACTCATTTTGTTCAGCTTTCCA-3′; *GAPDH*-F: 5′-ATGGAAATCCCATCACCATCTT-3′; *GAPDH*-R: 5′-CGCCCCACTTGATTTTGG-3′; *SNORD116-14*-F: 5′-TACATTCCTTGGAAAGCTGAACA-3′; *SNORD116-14*-R: 5′-TGGACCTCAGTTCGACGAGA-3′; *CD133*-F: 5′-AGTCGGAAACTGGCAGATAGC-3′; *CD133*-R: 5′-GGTAGTGTTGTACTGGGCCAAT-3′; *MDR1*-F: 5′-TTGCTGCTTACATTCAGGTTTCA-3′; *MDR1*-R: 5′-AGCCTATCTCCTGTCGCATTA-3′; *ESRP1*-F: 5′-GCCAAGCTAGGCTCGGATG-3′; *ESRP1*-R: 5′-CAGTCCTCCGTCAGTTCCAAC-3′. The miR-1206 primers and U6 primers were purchased from Ambion (Thermo Fisher Scientific, Waltham, MA, USA).

### 2.4. CCK-8 Assay

The proliferation of PC cells was evaluated using the CCK-8 assay (Dojindo, Kumamoto, Japan). Briefly, 1000 cells were cultured in 96-well plates and incubated for 96 h. To each well, 10 μL of CCK-8 reagents was added. The absorbance was measured at 450 nm. Using CCK-8 assay, we determined the viability of PC cells after treatment with gemcitabine for 72 h.

### 2.5. Subcutaneous Xenograft Experiments

Subcutaneous xenograft models of human PC were used to investigate the tumor formation ability of PC cells treated with or without gemcitabine, as previously reported [15]. All animal experiments were approved by the Animal Research Committee of Affiliated Tongji Hospital, Tongji Medical College, Huazhong University of Science and Technology (TJH-201901011). Four-week-old male BALB/c nude mice (*n* = 8 for each group) were purchased from Beijing HFK Bioscience (China). PaCa-2 cells transfected with the circ_0092367-expressing vector (or empty vector), or AsPC-1 cells transfected with LV-circ_0092367 (or LV-scramble), were subcutaneously injected into the right flank of nude mice. Seven days later, nude mice were treated with or without gemcitabine (50 mg/kg body weight, twice per week) intraperitoneally. Tumor volume (mm^3^) was calculated in the following way: tumor volume = (length [mm]) × (width [mm])^2^ × 0.5. Mice were sacrificed after 30 days. Tumor tissues were excised, and tumor weight was recorded.

### 2.6. Cell Invasion Assay

Matrigel invasion assay was used to assess PC cell invasion using the (Corning Costar Co, New York, NY, USA). In brief, PC cells were plated in the upper chambers with 500 μL serum-free media. The lower chamber contained RPMI-1640 medium (750 μL) with 10% FBS. Cells were cultured for 24 h. Then, invaded cells from random regions were fixed, stained with 0.1% crystal violet solution (Sigma, St. Louis, MO, USA), and counted using a microscope.

### 2.7. Western Blotting

Cells were lysed using a RIPA buffer (Beyotime Biotechnology, Beijing, China). Total proteins (30 µg) were separated by SDS-PAGE gels and then transferred onto PVDF membranes (Millipore, Burlington, MA, USA). The PVDF membranes were incubated with 5% non-fat milk in TBST buffer and were further incubated overnight at 4 °C with the primary antibodies (Cell Signaling, Danvers, MA, USA): E-cadherin, Vimentin, N-cadherin, ESRP1, cleaved caspase-3, MDR1, CD133, and β-actin. Membranes were incubated with secondary antibodies for 1 h at room temperature. The immune complex was detected by an ECL detection system (Amersham Biosciences, Buckinghamshire, UK).

### 2.8. Caspase-3/7 Activity Assay

The activity of Caspase-3/7 was determined using the Caspase-Glo 3/7 assay kit (Promega, Madison, WI, USA) according to the manufacturer’s protocol. PC cells were cultured in 96-well plates, and then 100 μL of Caspase-Glo 3/7 reagent was added to each well. Subsequently, cells were incubated for 1 h at room temperature, and luminescence was quantified in a luminometer (Victor, Perkin Elmer, Waltham, MA, USA).

### 2.9. Dual-Luciferase Reporter Assay

All luciferase reporter plasmids were obtained from Ribobio (Guangzhou, China). Mutations of the miR-1206-binding site in human circ_0092367 or *ESRP1* 3′-UTR sequence were created using a QuickChange XL Site-directed Mutagenesis kit (Stratagene, San Diego, CA, USA) according to A-G, T-C substitution criteria. The following primer sequences were used (circ_0092367-F: 5′-TTGTGTACTGTGCATTGTGAGGATGTATATTTGGTGCAGGTAGGCAGGCTTCCTCTCCAAACTGGG-3′; circ_0092367-R: 5′-CCCAGTTTGGAGAGGAAGCCTGCCTACCTGCACCAAATATACATCCTCACAATGCACAGTACACAA-3′; *ESRP1*-F: 5′-GGATAAAAACTCCACCAGTGTCTACCATCTCCACTGCAGGTTCTGTTAAGGAAGCTTCATTTTTGTATATTCCCG-3′; *ESRP1*-R: 5′-CGGGAATATACAAAAATGAAGCTTCCTTAACAGAACCTGCAGTGGAGATGGTAGACACTGGTGGAGTTTTTATCC-3′).

We transfected PC cells with wild-type (WT) or mutant (MUT) circ_0092367 luciferase vectors, WT or MUT *ESRP1* 3′-UTR luciferase vectors, the miR-1206 mimic or miR-1206 inhibitor, and the Renilla luciferase pRL-CMV vector (Promega, WI, USA). After 48 h of transfection, cells were harvested, and Firefly and Renilla luciferase intensity was detected using the Dual-Luciferase Reporter Assay System (Promega).

### 2.10. RNA Immunoprecipitation Assay (RIP)

RIP experiments were performed with a Magna RIP RNA-Binding Protein Immunoprecipitation Kit (Millipore, MA, USA). The magnetic beads were conjugated with an anti-Ago2 antibody (Millipore) or anti-IgG antibody (Millipore) and then incubated with cell lysates at 4 °C for 8 h. The protein was digested using proteinase K, and purified RNAs were used for the qRT-PCR analysis of circ_0092367 and miR-1206 expression.

### 2.11. Statistical Analysis

Results are shown as means plus or minus the standard deviation. The significance was calculated using Student’s *t*-tests or one-way ANOVA tests. Statistical significance was defined at *p* < 0.05.

## 3. Results

### 3.1. Circ_0092367 Expression Is Downregulated in PC

**Figure 1:**
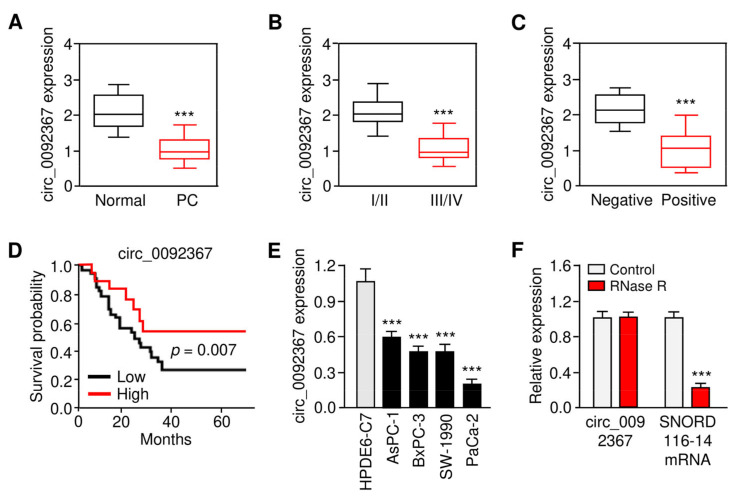
Circ_0092367 Expression Is Downregulated in PC. (**A**) qRT-PCR analysis of circ_0092367 expression in PC tissues compared with matched adjacent normal tissues. (**B**) The expression level of circ_0092367 in PC patients with different tumor stages. (**C**) The expression level of circ_0092367 in PC patients with or without lymph node metastasis. (**D**) Kaplan–Meier curves in PC patients with low versus high expression of circ_0092367. (**E**) qRT-PCR analysis of circ_0092367 expression in human PC cell lines and a normal pancreatic cell line HPDE6-C7. (**F**) Circ_0092367 and *SNORD116-114* mRNA expressions were analyzed with qRT-PCR assays in PaCa-2 cells treated with (or without) RNase. The experiments in (**E**) and (**F**) were repeated three times independently with similar results. *** *p* < 0.001.

First, we aimed to examine the expression and prognostic relevance of circ_0092367 in the development of PC. We used qRT-PCR assays to compare the expression of circ_0092367 in 40 pairs of PC tissues and their matched and normal tissues. Circ_0092367 levels were significantly decreased in PC samples compared with normal tissues (Figure 1A). A lower expression of circ_0092367 was correlated with an advanced tumor stage in PC (Figure 1B). Further analysis determined that the downregulation of circ_0092367 occurs more frequently in those patients with lymph node metastasis compared with the patients without metastasis (Figure 1C). The Kaplan–Meier survival curves revealed that the downregulation of circ_0092367 was associated with worse survival in patients with PC (Figure 1D). The qRT-PCR assays showed that, compared with HPDE6-C7 cells, the levels of circ_0092367 were significantly downregulated in four PC cell lines (Figure 1E). Furthermore, we found that circ_0092367 was more stable than linear SNORD116-14 mRNA (Figure 1F). The data suggest that circ_0092367 is frequently downregulated in PC tissue samples as well as PC cells, and decreased circ_0092367 expression is associated with an unfavorable outcome of PC patients.

### 3.2. Overexpression of Circ_0092367 Inhibits Xenograft Tumor Growth and Gemcitabine Resistance

**Figure 2:**
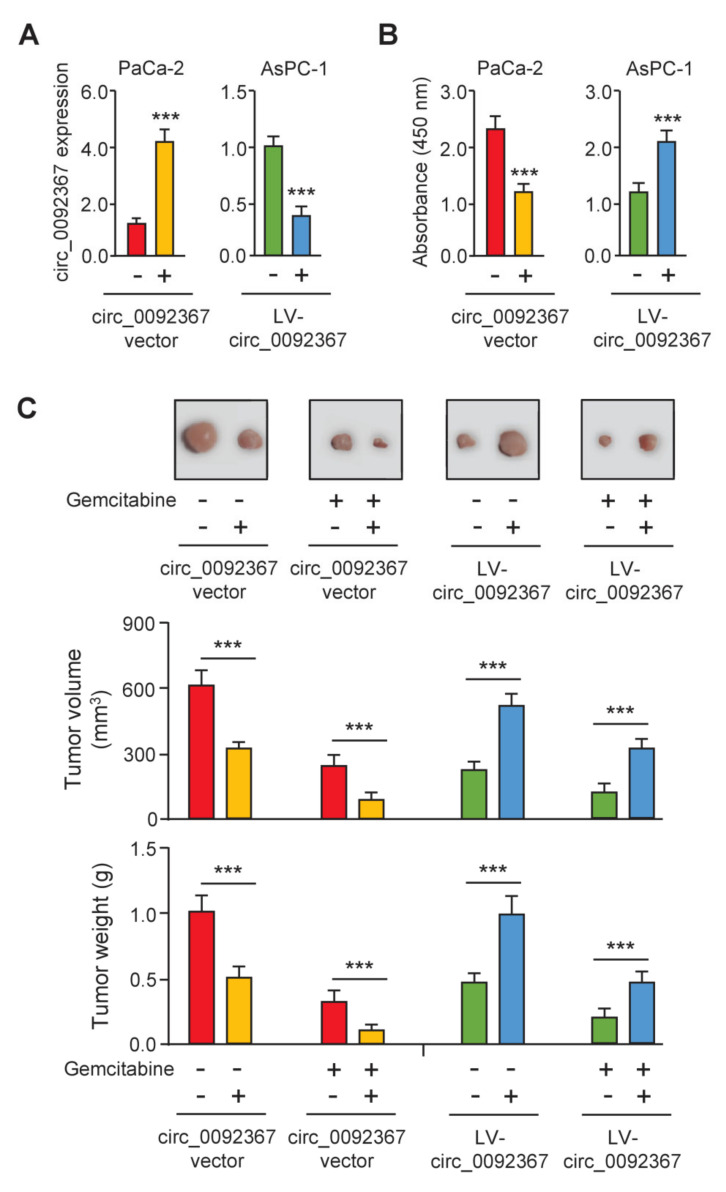
Overexpression of Circ_0092367 Inhibits PC Growth and Gemcitabine Resistance in Vivo. (**A**) Circ_0092367 expression in PaCa-2 cells transfected with circ_0092367-expressing vector or empty vector, or AsPC-1 cells after transfection of LV-circ_0092367 or LV-scramble, was assessed using qRT-PCR analysis. (**B**) CCK-8 assay in PC cells after overexpression or silencing of circ_0092367. (**C**) Xenograft tumor models were established using PaCa-2 cells transfected with circ_0092367-expressing or empty vector and AsPC-1 cells after transfection of LV-circ_0092367 or LV-scramble. Representative images of PaCa-2 or AsPC-1 tumors in nude mice treated with (or without) gemcitabine (upper). Tumor volume and tumor weight (bottom) were assessed for each derived xenograft tumor. The experiments in (**A**,**B**) were repeated three times independently with similar results. *** *p* < 0.001.

To reveal the functional roles of circ_0092367 in PC development and chemoresistance, we overexpressed circ_0092367 in the PC cell line PaCa-2, and also knocked down circ_0092367 expression in another PC cell line AsPC-1. The expression of circ_0092367 remarkably increased or decreased as expected (Figure 2A). CCK-8 assay showed that the upregulation of circ_0092367 suppressed cell proliferation, while reduced circ_0092367 expression led to opposite effects (Figure 2B). Then, we performed a tumorigenesis assay by injecting PC cells subcutaneously to nude mice. Tumor growth was significantly attenuated by circ_0092367 overexpression, and tumor growth was significantly enhanced by circ_0092367 knockdown (Figure 2C). Tumor volume and weight were suppressed following treatment with gemcitabine compared with those in the control group (Figure 2C). Notably, the overexpression of circ_0092367 increased the sensitivity of tumors to gemcitabine in vivo (Figure 2C). However, the silencing of circ_0092367 inhibited the effects of gemcitabine on PC growth in vivo (Figure 2C). Together, these data indicate that the overexpression of circ_0092367 inhibits PC growth and gemcitabine resistance in vivo.

### 3.3. Overexpression of Circ_0092367 Inhibits Invasion, EMT, and Gemcitabine Resistance in PC Cells

**Figure 3:**
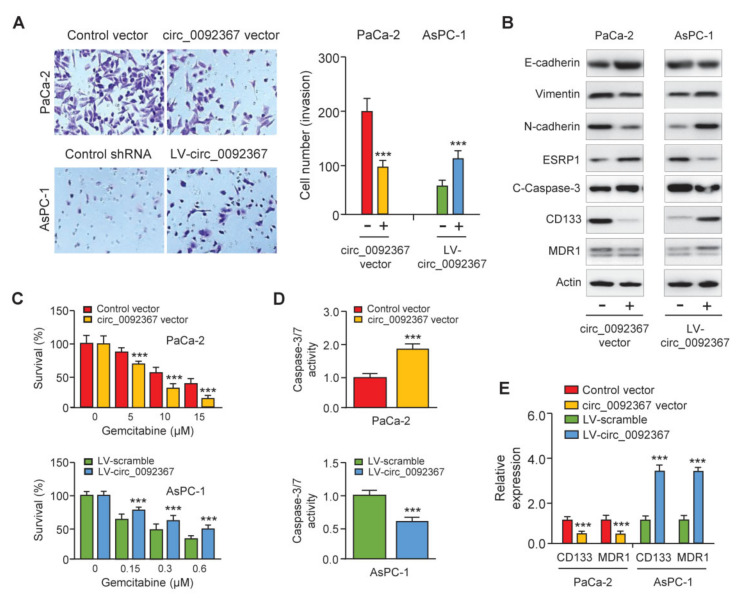
Circ_0092367 Represses Invasion, EMT, and Gemcitabine Resistance in PC Cells. (**A**) Invasion assay showed the effects of circ_0092367 on cell invasiveness. (**B**) Western blotting analysis was conducted to explore the expression of EMT-related proteins, cleaved-caspase-3, CD133, and MDR1 in PC cells following overexpression or knockdown of circ_0092367. (**C**) PC cells overexpressing or under-expressing circ_0092367 were treated with gemcitabine for different dilutions as indicated. A comparison of cell viability was performed using CCK-8 assays. (**D**) Effects of circ_0092367 on gemcitabine-induced cell apoptosis were detected using the Caspase-3/7 assay. (**E**) CD133 and MDR1 expressions in PC cells after either overexpression or silencing of circ_0092367 were measured using qRT-PCR analysis. The experiments in (**A**,**C**–**E**) were repeated three times independently with similar results. *** *p* < 0.001.

We performed cell invasion assays to study the function of circ_0092367 in PC cells. The overexpression of circ_0092367 significantly suppressed cell invasion, whereas the downregulation of circ_0092367 significantly increased cell invasion (Figure 3A). To identify whether circ_0092367 repressed cell invasiveness through regulating the EMT process, we examined the expression of several EMT-related genes in PC cells after the overexpression or knockdown of circ_0092367. Western blots showed that circ_0092367 overexpression induced E-cadherin expression, but decreased N-cadherin and Vimentin expression (Figure 3B). In contrast, the silencing of circ_0092367 had opposite effects in AsPC-1 cells (Figure 3B), indicating that circ_0092367 inhibits EMT in PC cells. To explore whether circ_0092367 can modulate the expression of cleaved-caspase-3, CD133, and MDR1 in PC cells, we performed Western blotting analysis and found that the overexpression of circ_0092367 in PaCa-2 cells increased the protein levels of cleaved-caspase-3, but reduced the expression of CD133 and MDR1. In contrast, the downregulation of circ_0092367 in AsPC-1 cells had opposite effects on the expression of these proteins (Figure 3B).

To further explore the possibility that the downregulation of circ_0092367 modulates the sensitivity of PC cells to gemcitabine treatment, PC cells with (or without) circ_0092367 overexpression (or knockdown) were treated with gemcitabine, and cell viability was investigated using CCK-8 assay. The sensitivity of PC cells to gemcitabine was significantly enhanced after the overexpression of circ_0092367 (Figure 3C). Moreover, AsPC-1 cells under-expressing circ_0092367 showed more resistance to gemcitabine treatment compared with the respective control cells (Figure 3C). The caspase-3/7 activity assays suggested that the overexpression of circ_0092367 increased apoptosis in PaCa-2 cells treated with gemcitabine (Figure 3D). After treatment with gemcitabine, apoptosis in AsPC-1 cells under-expressing circ_0092367 was much lower than in control cells (Figure 3D). Consistently, the qRT-PCR analysis showed that circ_0092367-overexpressing cells had lower levels of cancer stem-like marker CD133 and drug resistance-related gene MDR1 (Figure 3E). AsPC-1 cells under-expressing circ_0092367 showed a higher expression of CD133 and MDR1 (Figure 3E). Collectively, our results suggest that circ_0092367 represses invasion and EMT and inhibits gemcitabine resistance in PC cells.

### 3.4. Circ_0092367 Serves As a Sponge for MiR-1206 in PC Cells

**Figure 4:**
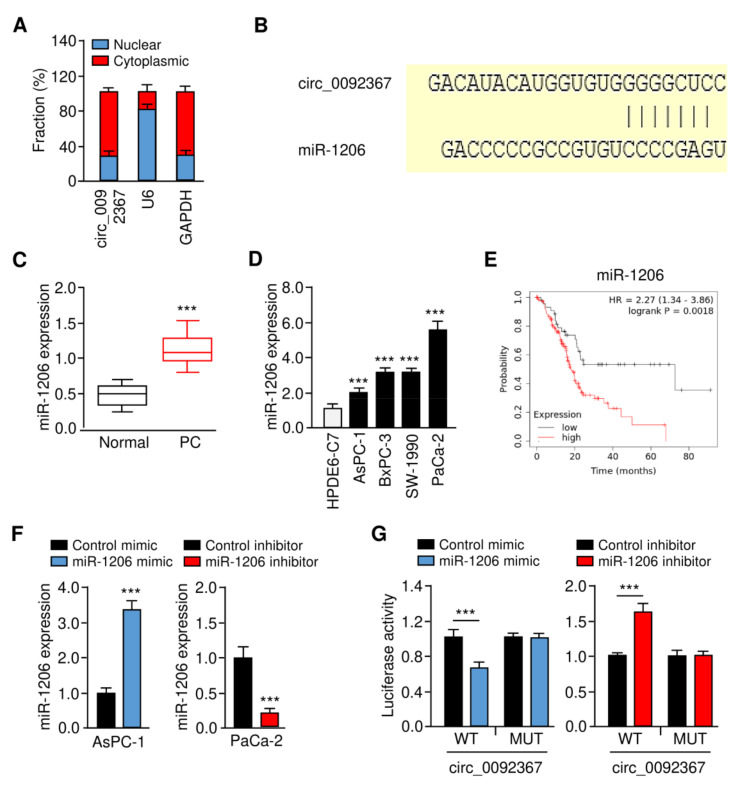
Circ_0092367 Serves as a MiR-1206 Sponge in PC Cells. (**A**) The subcellular location of circ_0092367 in PaCa-2 cells. (**B**) The complementary sequence between miR-1206 and circ_0092367 (CircInteractome). (**C**) MiR-1206 expression in PC tissues and adjacent normal tissues. (**D**) MiR-1206 expression in PC cells and normal cells. (**E**) Kaplan–Meier survival analysis of PC patients with lower versus higher miR-1206 expression. (**F**) MiR-1206 expression in PC cells transfected as indicated was investigated with qRT-PCR analysis. (**G**) PC cells were transfected with wild-type (WT) or mutant (MUT) circ_0092367, with miR-1206 mimic and miR-1206 inhibitor, and luciferase assays were performed. The experiments in (**A**,**D**,**F**,**G**) were repeated three times independently with similar results. *** *p* < 0.001.

To further explore the mechanism of circ_0092367 in PC pathogenesis, we analyzed the subcellular location of PC using qRT-PCR assays. Circ_0092367 was predominantly localized in the cytoplasm (Figure 4A). To find those miRNAs that may interact with circ_0092367, we predicted the miRNAs using the CircInteractome database. As a result, we found that miR-1206 had a complementary binding sequence to circ_0092367 (Figure 4B). Our qRT-PCR assays suggested that miR-1206 expression in PC tissues was much higher than normal tissues (Figure 4C). Additionally, miR-1206 was upregulated in PC cell lines compared to HPDE6-C7 cells (Figure 4D). Using the Kaplan–Meier Plotter database, we observed that increased expression of miR-1206 was associated with worse survival in PC patients (Figure 4E). To validate whether miR-1206 binds to circ_009236, we performed luciferase reporter assays. Relative to the control mimic, the miR-1206 mimic was able to reduce the luciferase activities of the WT circ_0092367 (Figure 4F,G). However, the silencing of miR-1206 could significantly elevate the luciferase activities of WT circ_0092367 (Figure 4F,G). By mutating the miR-1206 binding site in the circ_0092367 sequence, we observed that the transfection of either the miR-1206 mimic or miR-1206 inhibitor failed to impact the luciferase activity (Figure 4G).

**Figure 5:**
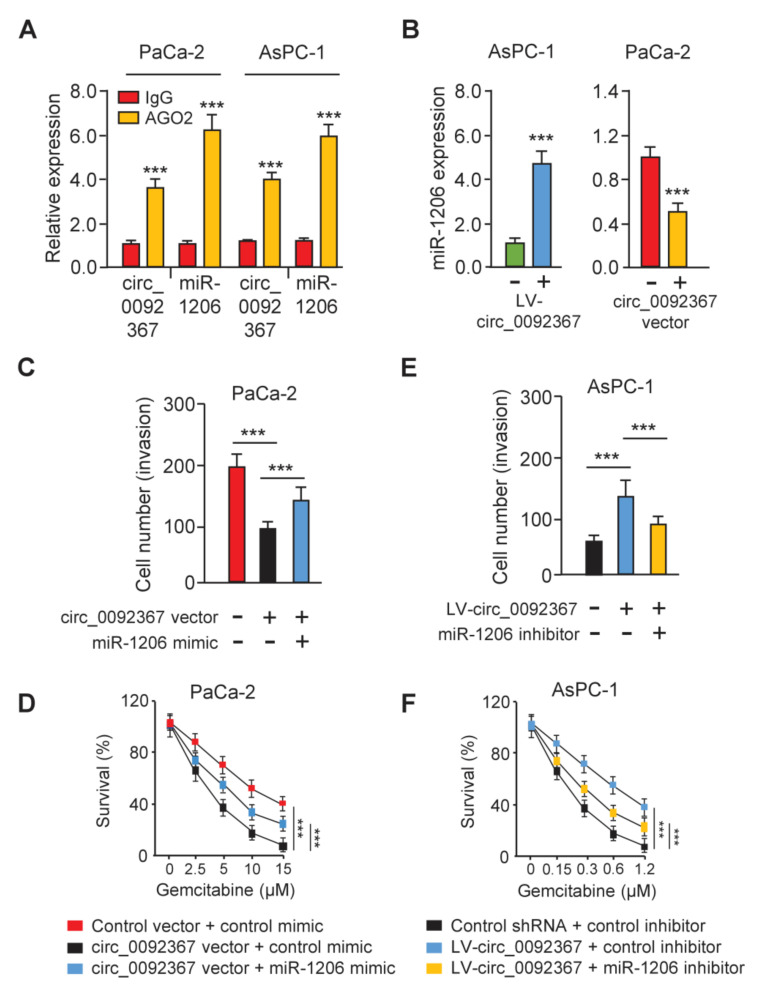
Circ_0092367 Sponges MiR-1206 in PC Cells. (**A**) RIP assay was used to quantify the expression of circ_0092367 and miR-1206 in PC cells. (**B**) miR-1206 expression in PC cells was tested after either overexpression or knockdown of circ_0092367. (**C**,**D**) Decreased cell invasion (**C**) and gemcitabine resistance (**D**) in circ_0092367-overexpressed cells were rescued by miR-1206 mimic. (**E**, **F**) However, increased cell invasion (**E**) and gemcitabine resistance (**F**) in circ_0092367-knockdown cells were reversed by miR-1206 inhibitor. The experiments in (**A**–**F**) were repeated three times independently with similar results. *** *p* < 0.001.

In addition, we conducted the RIP assay to extract circ_0092367 and miR-1206 with anti-AGO2 antibody PC cells. The results demonstrated that circ_0092367 and miR-1206 were pulled down by anti-AGO2 antibody, but not by anti-IgG (Figure 5A), supporting that circ_0092367 binds to miR-1206. Furthermore, we showed that the overexpression of circ_0092367 inhibited miR-1206 expression, while the downregulation of circ_0092367 induced miR-1206 expression (Figure 5B), suggesting that circ_0092367 sponges miR-1206 in PC cells. We also investigated whether circ_0092367 shows its tumor-suppressive role via sponging for miR-1206. Cell invasion assays and CCK-8 assays showed that the miR-1206 mimic could functionally restore circ_0092367 overexpression-suppressed cell invasion and gemcitabine resistance (Figure 5C,D). On the other hand, the miR-1206 inhibitor can significantly prevent circ_0092367 knockdown-enhanced cell invasion and gemcitabine resistance (Figure 5E,F). Therefore, circ_0092367 inhibits the invasion of PC cells and reverses the resistance of PC cells to gemcitabine through reducing miR-1206 expression.

### 3.5. Expression Pattern and Prognostic Value of ESRP1 in PC

**Figure 6:**
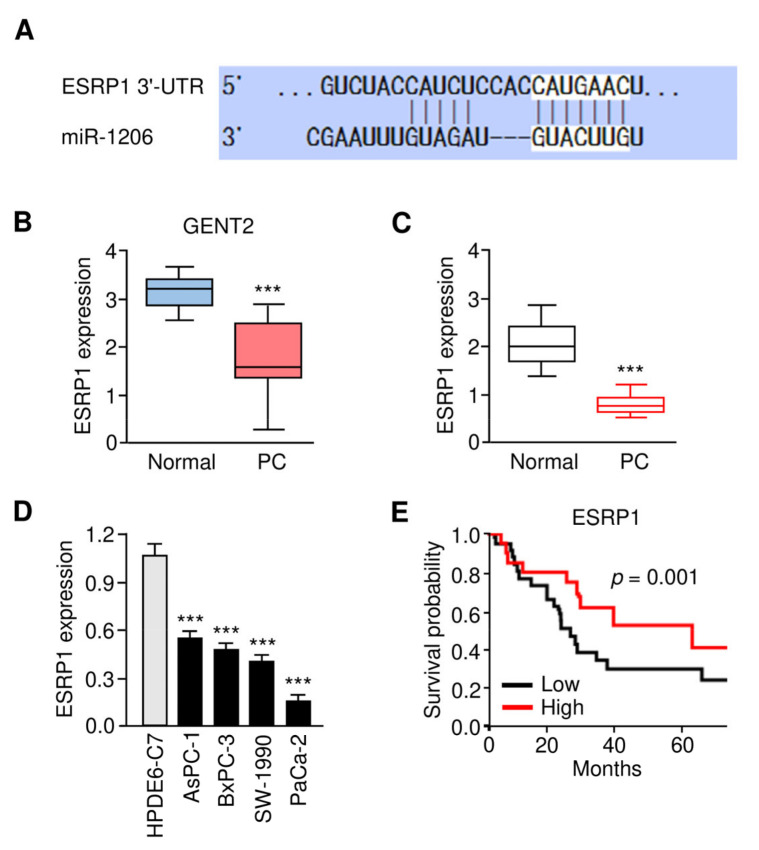
The Expression Pattern and Prognostic Significance of ESRP1 in PC. (**A**) The complementary sequence between miR-1206 and ESRP1 mRNA (TargetScan database). (**B**) ESRP1 expression in PC tissues and adjacent normal samples (GENT2 database). (**C**) *ESRP1* mRNA expression in PC tissues and normal samples was examined using the qRT-PCR assay. (**D**) qRT-PCR analysis of ESRP1 mRNA expression in PC cells and normal cells. (**E**) Kaplan–Meier survival analysis of PC patients with lower or higher *ESRP1* expression. The experiments in (**D**) were repeated three times independently with similar results. *** *p* < 0.001.

Based on our data that circ_0092367 suppresses the expression of miR-1206 to attenuate PC cell invasion and gemcitabine resistance, we, therefore, hypothesized that circ_0092367 could induce the levels of miR-1206 downstream genes. Using the TargetScan database, we showed that miR-1206 may target the 3′-UTR sequence of *ESRP1* mRNA (Figure 6A). Moreover, we used the GENT2 database to validate that the levels of ESRP1 were significantly downregulated in PC tissues compared to normal tissues (Figure 6B). Furthermore, we compared the expression of *ESRP1* in PC and normal tissues using qRT-PCR assays. The results showed that *ESRP1* expression was significantly decreased in PC tissues compared with normal samples (Figure 6C). We also found that ESRP1 levels were lower in PC cells compared with HPDE6-C7 cells (Figure 6D). The patients expressing a lower expression of ESRP1 displayed poorer survival (Figure 6E). Importantly, the overexpression of circ_0092367 induced the protein levels of ESRP1, and the silencing of circ_0092367 reduced the levels of ESRP1 in PC cells (Figure 3B). Together, circ_0092367 induces ESRP1 expression by working as a miR-1206 sponge.

### 3.6. MiR-1206 Enhances PC Cell Invasion and Gemcitabine Resistance by Targeting ESRP1

**Figure 7:**
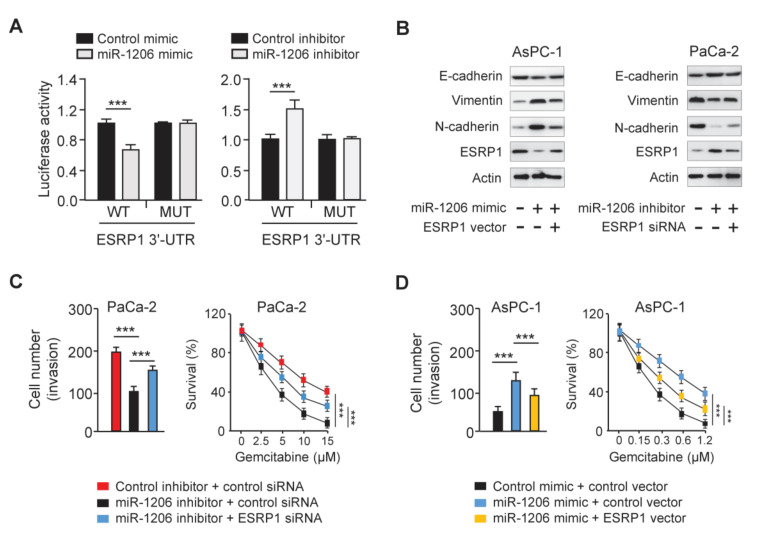
MiR-1206 Enhances PC Cell Invasion and Gemcitabine Resistance via Targeting ESRP1. (**A**) PC cells were transfected with either wild-type (WT) or mutant (MUT) *ESRP1* 3′-UTR, with miR-1206 mimic or miR-1206 inhibitor, and luciferase reporter assays were performed. (**B**) Protein levels of E-cadherin, N-cadherin, Vimentin, and ESRP1 in PC cells. (**C**,**D**) Invasion assays (left) and CCK-8 assays (right) in PaCa-2 (**C**) and AsPC-1 (**D**) cells. The experiments in (**A**,**C**,**D**) were repeated three times independently with similar results. *** *p *< 0.001.

To test whether miR-1206 directly regulates ESRP1 expression, we performed luciferase assays. Luciferase activities of the WT *ESRP1* 3′-UTR reporter vector were repressed by the miR-1206 mimic, and were elevated by the miR-1206 inhibitor (Figure 7A). Mutation of the miR-1206 putative binding sequence eliminated these effects (Figure 7A). Then, we examined the expression of the EMT-related genes and ESRP1 in AsPC-1 cells transfected with the miR-1206 mimic with (or without) the ESRP1 vector, and in PaCa-2 cells transfected with the miR-1206 inhibitor with (or without) ESRP1 siRNA. The results demonstrated that the overexpression of miR-1206 reduced E-cadherin expression, and induced N-cadherin and Vimentin levels (Figure 7B). However, the forced expression of ESRP1 reversed these changes (Figure 7B). Alternatively, the knockdown of miR-1206 induced E-cadherin levels, whereas it attenuated the levels of N-cadherin and Vimentin (Figure 7B). These changes were reversed by the transfection of ESRP1 siRNA (Figure 7B). We also performed rescue experiments and showed that miR-1206 knockdown decreased cell invasion and gemcitabine resistance, while the silencing of ESRP1 reversed these effects of miR-1206 (Figure 7C). MiR-1206 overexpression increased cell invasion and gemcitabine resistance, while these changes were largely reversed by the ectopic expression of ESRP1 (Figure 7D).

**Figure 8:**
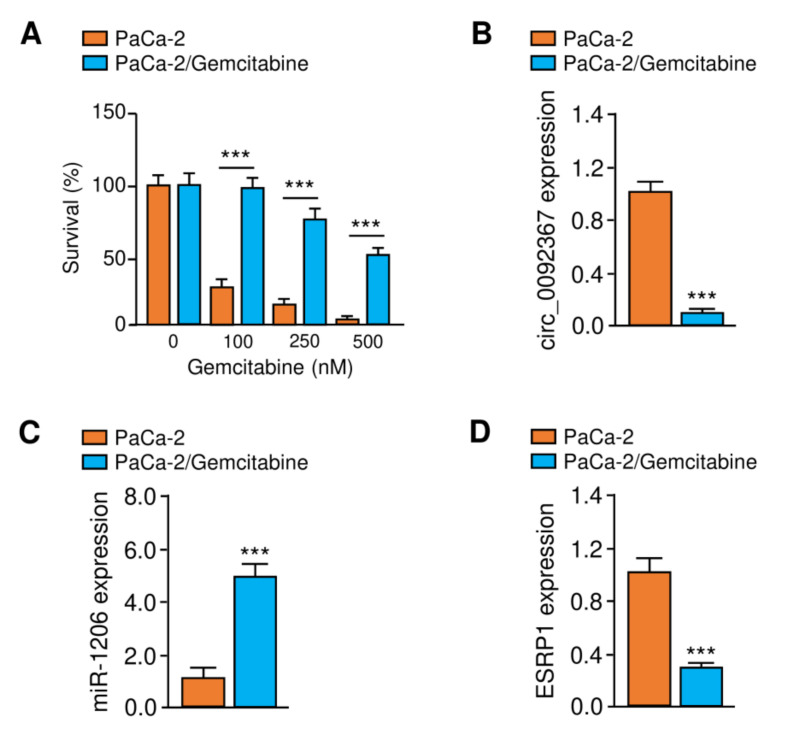
Expression of Circ_0092367, MiR-1206, and ESRP1 was examined in Gemcitabine-resistant PaCa-2 Cells. (**A**) CCK-8 assays were used to measure the effects of gemcitabine treatment on the survival of the parental cell line PaCa-2 and gemcitabine-resistant PaCa-2 cells. (**B**–**D**) Circ_0092367 (**B**), miR-1206 (**C**), and ESRP1 (**D**) expressions in gemcitabine-resistant PaCa-2/Gemcitabine cells and the parental PaCa-2 cell line were examined using qRT-PCR assays. The experiments in (**A**–**D**) were repeated three times independently with similar results. *** *p *< 0.001.

Gemcitabine-resistant cell line PaCa-2/Gemcitabine was generated by treating the parental PaCa-2 cell line with increasing concentrations of gemcitabine. CCK-8 assays showed that the gemcitabine-resistant PaCa-2 cells exhibit greater resistance to gemcitabine than the parental cells (Figure 8A). Consistent with our results shown above, gemcitabine-resistant PaCa-2 cells express lower levels of circ_0092367 and ESRP1 and higher levels of miR-1206 than the parental cells (Figure 8B–D). Collectively, these findings demonstrate that circ_0092367 inhibits EMT, PC cell invasion, and gemcitabine resistance via upregulating ESRP1 expression through sponging miR-1206.

### 3.7. Circ_0092367 Regulates the Expression of ESRP1 via MiR-1206

**Figure 9:**
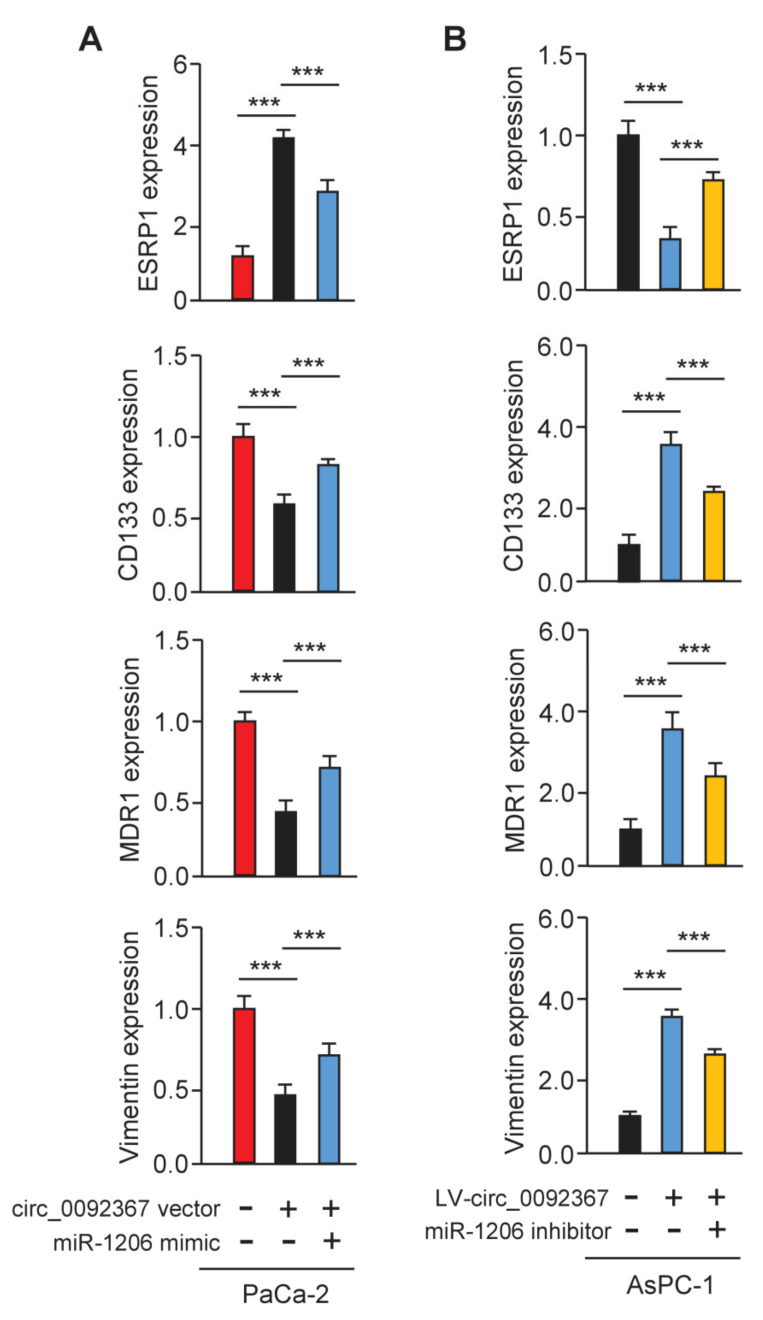
Circ_0092367 Regulates the Expression of *ESRP1* via MiR-1206. (**A**) qRT-PCR analysis of *ESRP1*, *CD133*, *MDR1,* and *Vimentin* levels in circ_0092367-overexpressing PaCa-2 cells overexpressing miR-1206 mimic and in control cells. (**B**) qRT-PCR analysis of *ESRP1*, *CD133, MDR1,* and *Vimentin* expression in circ_0092367-knockdown AsPC-1 cells with miR-1206 silencing and in control cells. The experiments in (**A**,**B**) were repeated three times independently with similar results. *** *p* < 0.001.

To examine whether circ_0092367 could mediate the expression of *ESRP1* through miR-1206, we conducted qRT-PCR experiments. As expected, *ESRP1* expression was greatly increased, while *CD133*, *MDR1*, and *Vimentin* levels were significantly reduced in circ_0092367-overexpressing PaCa-2 cells (Figure 9A). In addition, the knockdown of circ_0092367 significantly decreased the expression of *ESRP1*, but increased the expression of *CD133*, *MDR1*, and *Vimentin* (Figure 9B). The effects of circ_0092367 overexpression were reversed by transfection with the miR-1206 mimic, and circ_0092367 knockdown-induced effects were attenuated by the introduction of the miR-1206 inhibitor (Figure 9A,B). Therefore, the upregulation of miR-1206 is a critical mechanism by which circ_0092367 mediates its tumor suppressor roles in regulating the expression of ESRP1 and other EMT-related genes in PC cells.

## 4. Discussion

Gemcitabine is the first-line choice of chemotherapy approved for the treatment of PC patients [2]. However, most patients develop resistance to gemcitabine, and this resistance limits the efficacy of gemcitabine and leads to poor survival. EMT in PC cells drives gemcitabine resistance [16], yet the molecular events accounting for the acquisition of gemcitabine resistance are unclear. Recently, the dysregulation of miRNAs and circRNAs has been correlated with gemcitabine resistance [17,18,19]. To date, the functions and mechanisms of most non-coding RNAs in gemcitabine resistance remain undetermined. Here, we discovered for the first time that circ_0092367 sensitizes PC cells to gemcitabine treatment in vitro and in vivo. Subsequent experiments demonstrated that circ_0092367 regulates gemcitabine resistance through the miR-1206/ESRP1 axis.

Circ_0092367 expression was reduced in PC samples [13]. However, its biological roles and mechanisms in EMT and gemcitabine resistance are still unclear. In this study, we confirmed that circ_0092367 expression was downregulated in PC tissues, and its expression was negatively correlated with tumor stage and lymph node metastasis and positively associated with a favorable outcome of PC patients, demonstrating that circ_0092367 might be a potential prognostic biomarker for PC.

Increasing evidence suggests that circRNAs exert their regulatory roles across a range of cancer types [12]. CircRNAs have important roles in cancer progression and metastasis by regulating the hallmarks of cancer, such as proliferation, migration, and invasion [20]. In addition, circRNAs actively modulate EMT phenotypes through several mechanisms [21]. For example, circPRMT5 sponges miR-30c in bladder cancer to increase Snail expression [22]. Moreover, circPTK2 inhibits EMT and metastasis in lung cancer by disrupting the oncogenic functions of miR-429/miR-200b-3p [23,24]. CircNEK6 functions as a competing endogenous RNA of miR-370-3p in thyroid cancer, leading to the activation of Wnt/β-catenin signaling [25]. Our experiments showed that circ_0092367 inhibits EMT and gemcitabine resistance in PC cells. Therefore, we identified a new mechanism by which the downregulation of circ_0092367 resulted in EMT phenotypes during PC progression and chemoresistance.

CircRNAs could regulate gene expression by functioning as miRNA sponges [23,24,25]. To investigate the mechanism by which circ_0092367 mediates EMT and gemcitabine resistance, we performed luciferase assays and showed that circ_0092367 binds directly to miR-1206 and inhibits its expression. Therefore, our results were consistent with the competing endogenous RNA hypothesis, which proposes that circRNAs could competitively absorb miRNAs, relieving the effects of miRNAs on their target genes [12]. Given that circ_0092367 contains multiple miRNA-binding sites, we speculated that circ_0092367 might functionally act as a powerful sponge for some other miRNAs in PC cells.

PVT1 was shown to be an important oncogenic lncRNA that was overexpressed in many cancers, including PC [26]. In gemcitabine-resistant PC cells, PVT1 was upregulated and the overexpression of PVT1 promoted the resistance of PC cells to gemcitabine in vitro and in vivo [15]. The same study found that PVT1 sponges miR-619-5 to regulate Wnt/β-catenin signaling, therefore increasing gemcitabine resistance [15]. Importantly, PVT1 itself was processed into several oncogenic or tumor suppressor miRNAs (including miR-1206, miR-1204, miR-1207-3p, miR-1207-5p, and miR-1208) [26]. In this study, we had new findings showing that circ_0092367 suppressed EMT and sensitized PC cells to gemcitabine, but the introduction of miR-1206 reversed these effects of circ_0092367, suggesting a critical tumor-promoting role of miR-1206 in EMT and gemcitabine resistance. Considering this function of miR-1206, developing a miR-1206-targeted therapy might be encouraging for PC treatment.

Although ESRP1 has varying functions in each cancer type, it might be a metastatic suppressor or oncogene in a tissue-dependent manner [6,27,28]. In PC, ESRP1 has been verified as a favorable prognostic factor and a tumor suppressor [6,27]. Selective siRNA-mediated suppression of ESRP1 significantly increased PC cell growth, migration, and invasion [28]. Interestingly, the upregulation of ESRP1 was shown to reverse the EMT properties in PC cells [28]. To date, the roles and underlying mechanisms of ESRP1 in PC chemoresistance are still unknown. Here, we identified that the overexpression of ESRP1 could not only suppress EMT and cell invasion, but also sensitize PC cells to gemcitabine. Thus, our results underscore the importance of ESRP1 re-expression in eliminating EMT-derived mesenchymal cells and in overcoming gemcitabine resistance in PC cells.

## 5. Conclusions

**Figure 10:**
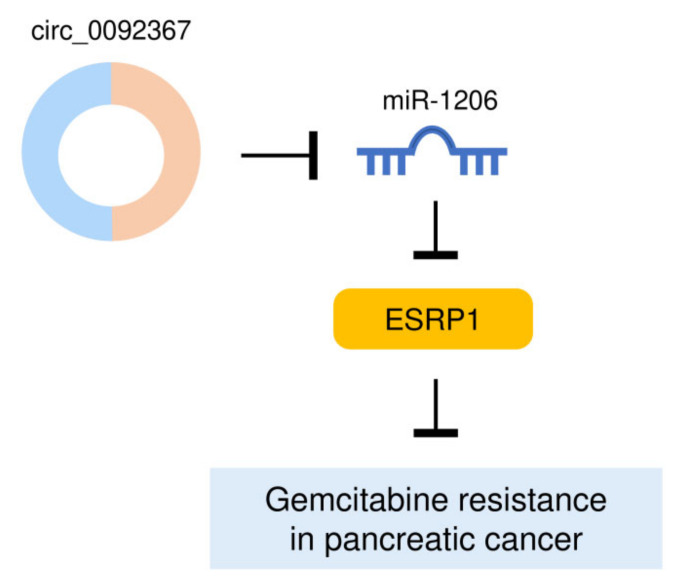
A Model Showing That Circ_0092367 Reverses PC Gemcitabine by Regulating the MiR-1206/ESRP1 Pathway.

Our findings identified that a decreased expression of circ_0092367 drives aggressive EMT properties in PC cells and promotes gemcitabine resistance via regulating the miR-1206/ESRP1 axis (Figure 10). These results would offer novel therapeutic targets, circ_0092367, miR-1206, and ESRP1, to expand the future treatment options for PC patients.

## Data Availability

The datasets analyzed in this study can be found at the Cancer Genome Atlas (TCGA) database (http://cancergenome.nih.gov).
